# Mechano-Signaling Aspects of Hepatocellular Carcinoma

**DOI:** 10.7150/jca.60102

**Published:** 2021-09-03

**Authors:** Mehak Passi, Stefan Zahler

**Affiliations:** Center for Drug Research, Ludwig-Maximilians-University, Butenandtstr. 5-13, 81377 Munich, Germany.

**Keywords:** Hepatocellular carcinoma, HCC, mechano-signaling, extracellular matrix, ECM, stiffness

## Abstract

HCC is one of the leading causes of cancer related death worldwide and comprises about 90% of the cases of primary liver cancer. It is generally accompanied by chronic liver fibrosis characterised by deposition of collagen fibres, which, in turn, causes enhanced stiffness of the liver tissue. Changes of tissue stiffness give rise to alterations of signalling pathways that are associated to mechanical properties of the cells and the extracellular matrix, and that can be subsumed as “mechano-signaling pathways”, like, e.g., the YAP/TAZ pathway, or the SRF pathway. Stiffness of the liver tissue modulates mechanical regulation of many genes involved in HCC progression. However, mechano-signaling is still rather underrepresented in our concepts of cancer in comparison to “classical” biochemical signalling pathways. This review aims to give an overview of various stiffness induced mechano-biological aspects of HCC.

## Introduction

Hepatocellular carcinoma (HCC) accounts for 90% of all cases of primary liver cancer and is the third leading cause of cancer-related death worldwide 1. Every year the incidence of occurrence of HCC in Europe and the United States increases, and currently accounts for more than 626,000 cases worldwide. Many factors such as hepatitis B virus (HBV), hepatitis C virus (HCV), alcohol and non-alcoholic steatohepatitis (NASH) contribute to the development and progression of HCC. Further, several point mutations, such as copy number aberrations (CNAs), insertions and deletions, virus integrations and gene fusions have been reported in the context of HCC 2. Their role as drivers, however, is still unclear.

More than eighty per cent of HCC cases are associated with fibrosis induced by chronic liver injury and develop in fibrotic or cirrhotic liver. HCC is associated with the differentiation of hepatic stellate cells (HSCs) into myofibroblasts, which produce extracellular matrix (ECM). Fibrosis is generally considered as a protective response to acute liver injury and results in the replacement of injured liver parenchyma with scar tissue. Fibrosis becomes chronic and recurring if the hepatocellular injury persists. However, the role of fibrosis in promoting HCC has not been clearly established to date 3.

Generally, fibrosis is characterized by deposition of collagen fibrils of varying degree (mostly type 1 collagen) in the fibrotic liver, resulting in up to 5-fold increase of total collagen content. Fibrosis can vary from mild to bridging fibrosis and cirrhosis depending on the degree of collagen deposition. Moreover, hepatic ECM consists of many other proteins, such as non-collagenous glycoproteins (elastin, fibronectin and laminin), matricellular proteins (thrombospondins, osteopontin, tenascins, and members of the clathrin coated vesicle protein family) and connective tissue growth factor (CTGF). During liver fibrosis, many of these proteins undergo biochemical alterations 3, 4.

All these changes result in overall modulation of the hepatic extracellular matrix (ECM). They are a result of various mechanical and biochemical feedback loops that play an important role in modifying the hepatic ECM. Moreover, receptor independent mechanosensitive signal transduction pathways, such as YAP/TAZ, are involved in controlling cell survival and differentiation, and are activated by stiff ECM. The activation of the YAP/TAZ is caused via an upstream inhibition of Hippo pathway kinases on stiff substrates. Apart from the Hippo pathway, other pathways, such as Wnt/β-catenin, PTEN and the Notch signaling pathway also contribute to HCC progression 5. Therefore, studying the role of mechanics is an important aspect of HCC when it comes to its pathogenesis.

This review focuses on discussing and highlighting various mechano-sensing processes and signaling pathways involved in the occurrence and progression of HCC. It also highlights the importance of ECM stiffness in HCC and into how it is linked to mechanosensing in HCC.

## Extracellular Matrix stiffness and Hepatocellular carcinoma

During HCC there is a transition from the premalignant environment (PME) to the tumor microenvironment (TME) and the ECM undergoes a variety of changes - many of which are related to an alteration of mechanical properties. ECM not only provides mechanical strength but also regulates a variety of signaling cascades through its ability to bind to a wide variety of specific receptors such as integrins, growth factors, as well as to regulate their expression, distribution and activation 6. The changes in the ECM components such as collagens, glycosaminoglycans, laminins, proteoglycans, and fibronectin, result in overall behavioral and phenotypic changes in the epithelial, tumor and stromal cells 6, 7. With the help of transmembrane receptors (integrins, DDRs) surrounding cells sense these changes and regulate specific signaling pathways in response to the external stimuli 8, 9. Increased expression of integrins and DDR2 triggers a wide variety of signaling cascades, such as phosphoinositide 3 kinase (PI3K), mitogen-activated protein kinase (MAPK) and regulates EMT of HCC 10.

Reorganization and enhanced overproduction of ECM by myofibroblasts result in the mechanically stiff microenvironment that promotes tumor cell proliferation, invasion and changes in gene expression of stromal cells by enhancing tumor progression 11, 12. Tumor cells regulate matrix stiffness by not only influencing the degree of fibrosis but also by controlling cross-linking and expression of ECM proteins and certain enzymes such as lysyl oxidases 13, 14. Hepatic stellate cells (HSCs) also play a major role in remodeling and maintaining ECM through secretion of major ECM proteins such as collagen, and ECM degrading enzymes called matrix metalloproteinases (MMPs) 15. In the healthy liver, HSCs are in the quiescent state with cytoplasmic vitamin A droplets while following liver injury they lose their vitamin A droplets, and become highly activated and proliferative 16. They cause increased production of ECM components (collagen) and other ECM proteins. They are also associated with the increased inflammation signaling and matrix degradation which all together contribute to fibrosis. In HCC, increased stiffness leads to activation of HSCs followed by the activation of MMPs and tissue inhibitor of metalloproteinases (TIMP) 17, 18.

Quiescent HSCs balance the production of ECM proteins and MMPs proteolytic activity and maintain the ECM. As a consequence of liver injury HSCs get activated causing excess collagen production and matrix degradation leading to a stiff fibrotic state 19.

In normal liver where resolution of fibrosis occurs, increased activity of MMPs leads to collagen degradation and ECM softening following the reversal of activated HSCs to their quiescent phenotype. Whereas, conversely, in the stiff state of a fibrotic liver altered characteristics of the environment such as stiffness amplify the activated HSCs phenotype further contributing to fibrosis, and later HCC development 20, 21. Many signaling pathways in HSCs are highly mechanosensitive such as the HNF4α transcriptional network or integrin-mediated YAP activation 22, 23. Rigidity occurring from fibrosis may affect the ECM remodeling at the protein expression and secretion level that is mediated by HSCs. Two key MMPs secreted from HSCs are MMP-2 and MMP-9 that degrade collagen 24. Composition of ECM in fibrosis is also dependent on the sensitivity of the secretion of MMPs and TIMP (Tissue inhibitors of matrix metalloproteinase) by HSCs towards rigidity. It is known that fibrotic stiffness downregulates MMP-9 expression and secretion, and upregulate the secretion of TIMP-1 25.

Liver stiffness to some degree correlates with increased risk of HCC development. The Young's elastic modulus E for a healthy liver ranges between 300 and 600 Pa whereas in case of fibrosis and cirrhosis development it can be 20 kPa and higher 26. In some studies, it was found that patients with liver stiffness values of >12.5-13 kPa had a 4 to 13 fold increased risk of developing HCC 27. Changes in matrix stiffness modulate the behavior of epithelial cells through various mechanosensitive oncogenic pathways. They also contribute to the transformation and dedifferentiation of hepatocytes, and, in addition, lead to ductular and progenitor expansion in the liver, which in turn leads to HCC. Matrix stiffness provides a niche for tumor-initiating cells (TICs) and contributes to their proliferation by a variety of mechanosensing pathways. HSCs seem to differentiate into cancer-associated fibroblasts (CAFs) under mechanical stimuli, thereby providing a permanent feed-forward loop that continues to establish a stiff tumor niche 28, 29. This process of creation of premalignant microenvironments (PME) and the tumor microenvironment (TME) by CAFs has been reviewed very recently elsewhere 30. Intriguingly, CAFs have recently been proposed as therapeutic target for nanocarriers 31.

Expression of LOXL (Lysyl oxidase) has been identified as an important factor controlling ECM stiffness 32. It is upregulated by TGFβ 32, hypoxia-inducible growth factor 1 α (HIF-1α) 33, and increases stiffness by crosslinking ECM proteins. In turn, its expression increases stiffness 34, causing a positive feedback loop. In some studies, it has been shown that the inhibition of LOXL2 with a monoclonal antibody reduces stiffness, collagen deposition and tumor size by reducing fibroblast activation and fibrosis in the liver 34. However, NASH-induced fibrosis was not reduced in large scale clinical trials 35. Apart from the overproduction or biochemical alteration of ECM several other factors, such as high interstitial pressure as a result of hypervascularization, hyperproliferation, or cell swelling induced injury and inflammation contribute to increase stiffness of the tumors 36.

Increased stiffness promotes proliferation of cells, increases EMT (Epithelial-Mesenchymal Transition) and resistance to apoptosis, as well as the stemness of HCC cell lines 37. At the molecular level, increasing stiffness activates multiple signaling pathways such as YAP/TAZ, β-catenin pathway, PI3K/AKT, JNK, ERK, focal adhesion kinase pathway, finally resulting in enhanced HCC proliferation and chemotherapeutic resistance 38-40. The mechanical signaling pathways unfortunately are in part redundant and deeply interwoven, making their understanding and elucidation quite complex.

## Mechanotransduction in HCC induced by ECM-stiffness

The fibrotic program in HCC is generally accomplished by fibroblast mechanosensing of the stiffened ECM. Like in other cells, mechanosensing primarily is accomplished by the activation of integrins. Integrins get activated by binding of specific ligands leading to conformational changes and the formation of focal adhesion complexes. Several adaptor proteins such as paxillin, vinculin and talin facilitate the connection of integrin to the actin cytoskeleton, translating the mechanosensitive information into changes in cell contractility and mechanoresponsive signals. Strain stiffening driven by cell contractility of ECM causes elevated levels of integrin activation, leading, e.g., to phosphorylation of the kinases Src and FAK, and thus their activation 41-43. Increased stiffness stimulates HCC proliferation by inducing resistance towards Sorafenib through activation of β1 integrin/FAK signaling and enhancing nuclear translocation of YAP1. All these events result in the maturation of focal adhesion complexes and further activate a multitude of pathways such as MAPK, PI3K/AKT and YAP/TAZ 44, 45. The levels of total and phosphorylated FAK are increased in HCC and are generally related to vascular invasion, tumor stage and intrahepatic metastasis 46. Several compounds targeting FAK are under clinical and preclinical trials as specific deletion of FAK has been shown to reduce HCC proliferation and tumor-induced overexpression of cMET and β-catenin 47. By RNA sequencing it was found that increased stiffness activates hepatic stellate cells (HSCs), leading to the release of a set of paracrine factors, including CXCL12, IL6, IL11, PDGFA and B and VEGFA 48, which all promote colorectal liver metastasis in mice by paracrine mechanisms. However, it still remains unclear, whether liver fibrosis and stiffness promote malignant transformation of hepatocytes primarily through the effect on HSCs and the hepatic tumor microenvironment 49.

Agrin a heparin sulfate proteoglycan has recently been discovered to modulate the activation of YAP, and acts as a mechanotransduction signal in HCC. It is produced by endothelial, myofibroblast, and tumor cells, and is highly expressed in human cirrhotic liver and HCC 50. Knockdown of Agrin reduces proliferation, migration and invasion of tumor cells, and reduces the levels of EMT markers, while it induces apoptosis *in vitro*, as well as oncogenic signals and tumor growth in mice 51, 52. It has been shown that Agrin activates FAK-ILK-PAK1 pathways and transduces matrix rigidity through an integrin-Lrp4/MuSK pathway, resulting in activation of YAP in HCC 53. Agrin acts as a contributor to ECM sensing in HCC cells.

Serum Response Factor (SRF) is part of another well characterized mechanotransduction pathway. It is mediated by myocardial-like proteins (MRTF), induced by F-actin polymerization. Interestingly, SRF is not abundantly expressed by normal healthy hepatocytes and non-tumoral tissues. In contrast, in cells from high-grade human HCC and human HCC cell lines, it exhibits a strong nuclear expression 54. SRF target gene signatures partially overlap with the mechanical stress-induced signatures from YAP/TAZ target genes. Activation of SRF in hyperproliferative nodules of hepatocytes results in HCC development 55. On a molecular level overexpression of SRF promotes HCC cell invasion and migration by increasing expression of β-catenin and EMT genes 56, 57.

**Figure 1:**
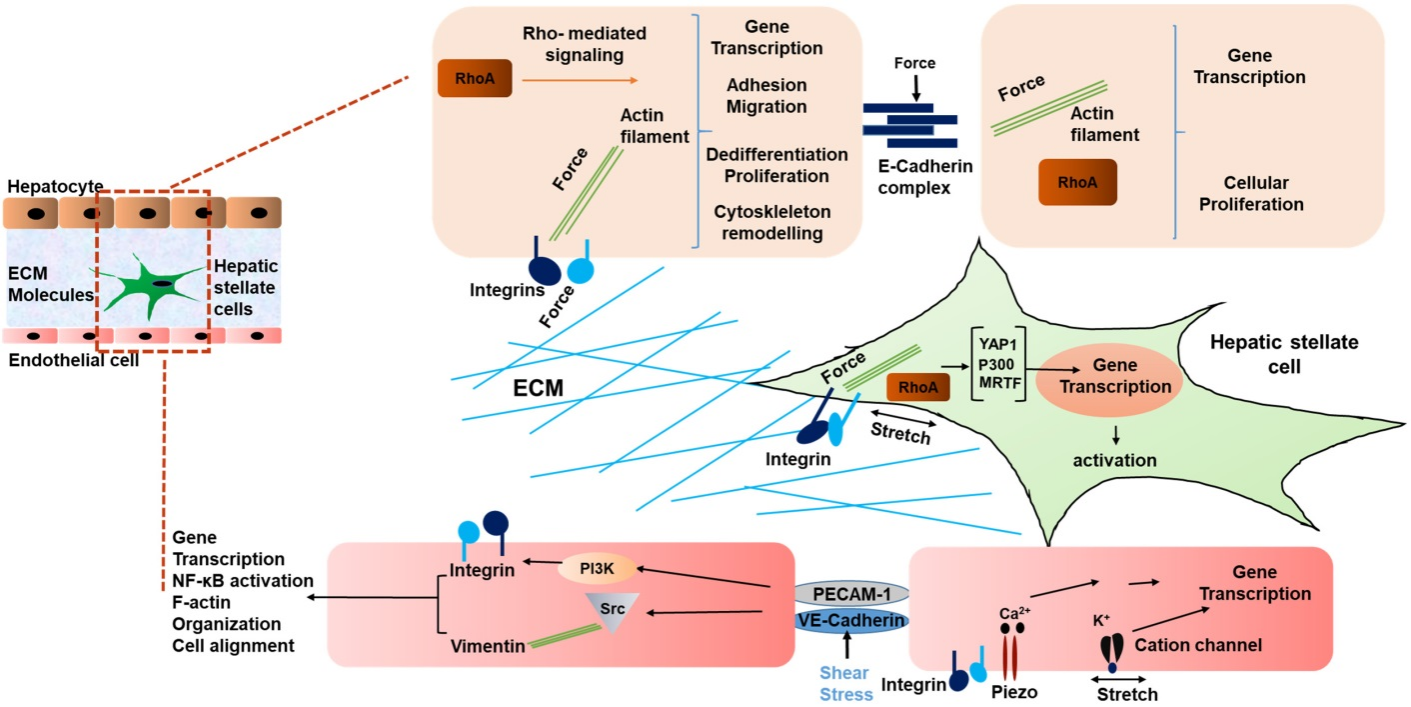
** Schematic representing mechanical signaling in HCC.** Mechanical forces are communicated by the ECM via integrins of hepatocytes and HSCs, and between cells via E-cadherin of hepatocytes. In liver sinusoidal endothelial cells, VE-cadherin and PECAM-1 communicate shear stress between cells, causing activation of integrins. In addition, cation channels are activated by mechanical stretch on LSECs 42. Thus, a multicellular network of force induced signaling is established.

Interestingly, not only HSCs, but also liver sinusoidal endothelial cells (LSECs) have been shown to respond to stiffness and mechanosensation 58. In a study by Liu et al, LSECs were embedded on 2D substrates underlying 3D collagen type 1 with integrated HSCs on the top. This was done to mimic the interaction of LSEC/HSC LSECs on substrates with stiffness values ranging from 140 Pa to 610 Pa. The LSECs formed capillary like structures under this condition. During the early phase of liver fibrosis angiogenesis is stimulated by the condensation of collagen fibers and generated forces by collagen remodeling that result in activation of collagen-DDR2-JAK2/P13K/AKT signaling promoting HSC activation 59. Thus, mechanical forces in liver generated due to fibrosis indeed influence the phenotype of LSECs, and in turn contribute to the development and progression of HCC 60, 61. Fig. 1 summarizes the various mechanical inputs related to development of HCC. All in all, the mechanical inputs described above converge at the modulation of transcriptional regulation. Table 1 summarizes genes mechanically regulated in liver disease.

## The Hippo/YAP/TAZ pathway

The Hippo signalling pathway plays a major role in the progression and pathogenesis of HCC, linking stiffness and mechanotransduction to cancer progression. The YAP/TAZ axis seems to be a hub, where all kinds of mechanosensitive processes converge. Therefore, we will occupy ourselves with this specific pathway in greater detail. Its major components and downstream effectors are YAP and TAZ. These are involved in many autonomous functions, such as cell proliferation, differentiation, survival, development, metabolism, and cross-talk with the immune system. The role of Hippo signalling in regulation and biological functions varies from organ to organ, and between specific cell types, making it even more complex to understand. Overexpression of activated YAP results in rapid development of HCC, confirming its potential oncogenic role 62, 63.

The activity of YAP and TAZ is controlled by MST1/2 kinases. These are upstream regulators of the Hippo pathway that restrict tissue overgrowth, size and carcinogenesis by regulating YAP and TAZ activation 64. YAP was the first protein identified with a WW domain (a motif comprising of 2 tryptophan residues), and is the key transcriptional regulator of the Hippo pathway, while TAZ is a YAP paralog (44% identity to YAP) 65, 66. In general, MST1/MST2 kinases activate the kinases LATS1 and LATS2 by phosphorylating them at Thr1079 and Thr1041, respectively. They also phosphorylate MOB 1A and 1B kinases at Thr35 and Thr12. MOB1A/MOB1B when activated interact with LATS1 and LATS2 and lead to the autophosphorylation of LATS1 and LATS2 67. Both of these phosphorylation events lead to the activation of the LATS1 and LATS2 kinases. LATS kinases - when activated further - phosphorylate YAP, leading to the cytoplasmic sequestration of YAP/TAZ or ubiquitin mediated protein degradation 68, 69.

**Figure 2:**
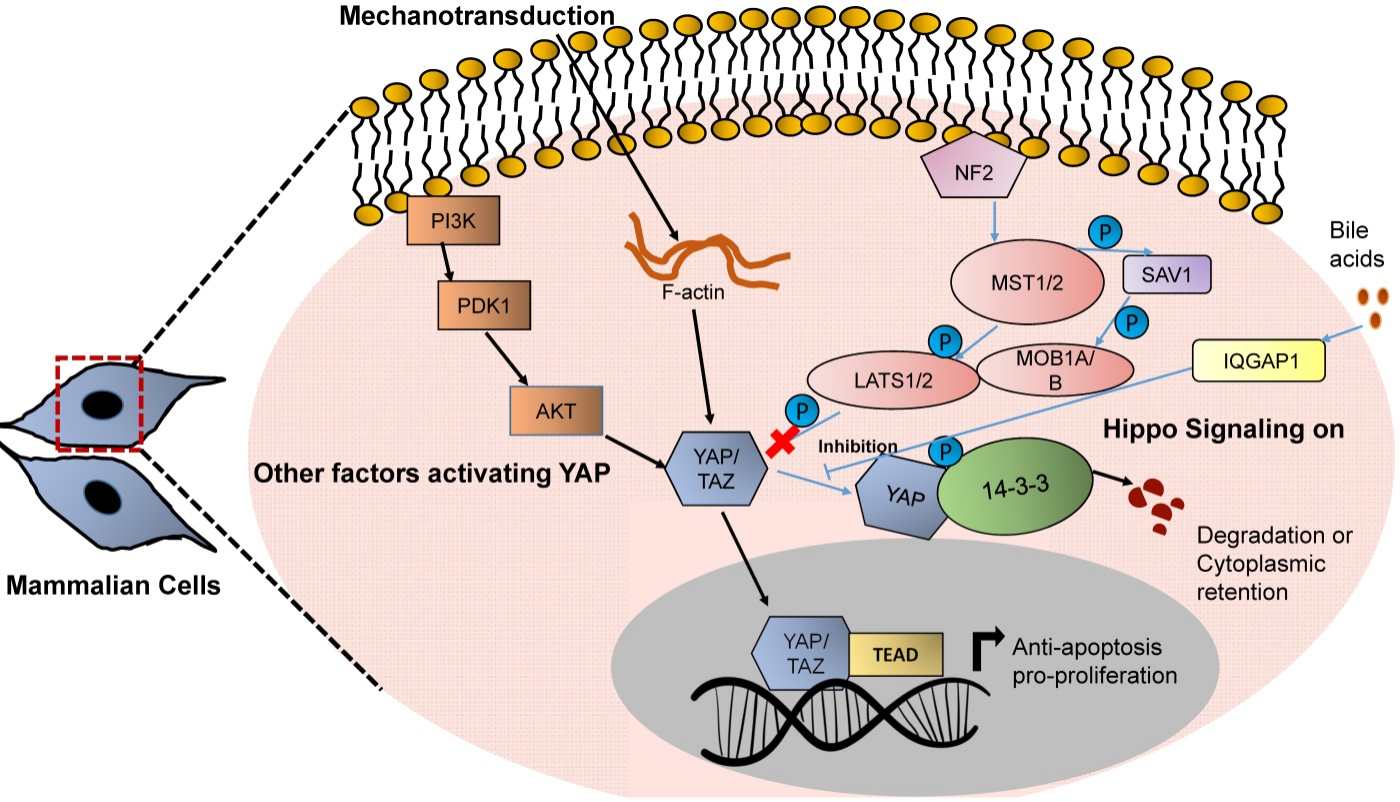
** YAP based mechanotransduction in HCC.** Increased stiffness activates the YAP based Hippo signaling network. YAP/TAZ when activated, gets translocated in the nucleus and regulates the transcription of genes related to proliferation and cellular growth.

When LATS kinases are inactive, YAP/TAZ is not phosphorylated and gets translocated in the nucleus where it binds to members of the TEAD family of transcription factors, and mediates expression of target genes, which are involved in cell growth, migration, proliferation and survival, such as cysteine-rich angiogenic inducer 61 (CYR61), connective tissue growth factor (CTGF), and others 70-74 (Fig. 2). RUNX3 physically interacts with the N-terminal region of TEAD through its Runt domain. This interaction markedly reduces the DNA-binding ability of TEAD that attenuates the downstream signaling of the TEAD-YAP complex. This transcription complex also interacts with Runt-related transcription factor 3. DNA-binding ability of TEAD is reduced markedly through this interaction, which attenuates the downstream signaling of TEAD-YAP 75, 76. In addition, YAP/TAZ also interacts with SMAD1, SMAD2/3 forming protein complexes suggesting a cross talk between the Hippo signaling pathway and the TGF-β pathway 77-79. YAP directly interacts with the p53 promotor to enhance its expression which results in p53- dependent cycle arrest and apoptosis. It furthermore induces p21, TBox and Caspase 3 expression and inhibits the expression of anti-apoptotic factors, such as Bcl-2 and Bcl-xl. It has recently been shown that LATS1/LATS2 contributed to the tumor suppressive feature of p53 under basal conditions 80, 81. YAP/TAZ also interact with the intracellular growth domain (ICD) of ErbB4 (one of the members of epidermal growth factor receptors in the nucleus) 82. All these events modulate the expression of genes involved in proliferation, differentiation, development and growth. Loss of any of the core components of the Hippo pathway such as MST1/MST2, LATS1/LATS2, MOB1A/MOB1B, etc., results in upregulation in target gene transcription of YAP/TAZ-TEAD further leading to uncontrolled cellular proliferation and tissue growth 83-85.

Due to fibrosis, mechanical strain occurs in the cirrhotic liver that activates YAP and TAZ, which in turn upregulate the expression of various target genes that control cellular proliferation and growth. YAP/TAZ are commonly overexpressed after deletion of Mst1/2 in hepatocytes and act as a transducer of liver tumor development and HCC 86, 87. A major pathway of activation of YAP/TAZ, however, is a loss of phosphorylation, and, consequently, nuclear translocation of these proteins. Force transduction from the extracellular space to intracellular signaling pathways via the actin cytoskeleton is responsible for the YAP and TAZ mechanotransduction. Many mechanosensory proteins such as integrins, adherens junctions, adaptor proteins such as vinculin and talin, SRC family kinases, as well as FAK and Rho-GTPases participate in activation of YAP/TAZ. Inhibitors of actin polymerization abolish YAP/TAZ mechanotransduction 88, 89. Overexpressing the activated form of TAZ in combination with the NRAS G12V mutation increases the formation of liver tumors, however to a lower degree than overexpressing activated YAP 90. A list of genes regulated by YAP and upregulated in HCC can be found in Table 2.

## Role of YAP and TAZ in HSCs

Hepatic stellate cells (HSCs) are one of the major drivers of liver fibrosis, and are also involved in the process of liver repair after acute liver injury 91. A fibrotic scar is formed during chronic liver injury after stellate cells are activated by excessive accumulation of ECM proteins, and trans-differentiation of quiescent HSCs into myofibroblasts. This process is generally controlled by the molecular drivers that regulate HSC activation. The Hippo signalling pathway has been recognized as one of the important pathways in stellate cell activation. During the acute liver regeneration process after hepatectomy, or ischemia- reperfusion injury and after chronic livery injury or CCl4 induced liver damage, YAP is activated in HSCs 92-94. Sustained activation of YAP in liver fibrosis is generally due to an increase in ECM protein levels, as well as tissue stiffness 95. In addition to HSCs, portal fibroblasts are also mechanosensitive. When seeded on polyacrylamide hydrogels both these cell types respond to increased stiffness 96-98. Immunohistochemical analysis of murine and patient samples show that more YAP is translocated into the nucleus from cytoplasm of the activated HSCs/myofibroblasts of fibrotic livers compared to normal livers. Nuclear localization of YAP (i.e., activation) is increased when HSCs are cultured on stiff plastic surfaces for 10 h. Moreover, inhibition of YAP activation by verteprofin *in vitro* mitigates stiffness mediated HSC activation. All these data suggest that YAP plays an integral role in stiffness mediated HSC activation 99. Furthermore, YAP and its transcriptional targets are upregulated in HSCs after 15 minutes of murine partial hepatectomy, as a result of elevated blood flow and shear stress, exerting mechanical forces. When stretching forces are applied to HSCs *in vitro*, HSCs are stimulated to produce fibronectin and also to promote fibril assembly by a β1-integrin/actin dependent mechanism 100. YAP phosphorylation and its cytoplasmic retention has been shown to be promoted by the loss of β1 integrin in HSCs 101, 102.

Megakaryoblastic leukaemia factor-1 (MKL1), also known as myocardia related transcription factor (MRTFA), is another transcriptional regulator that responds to force changes. It is generally bound to cytoplasmic G-actin and force mediated actin assembly leads to nuclear translocation 103, 104. Interestingly, expression of MKL1 and YAP targets is mutually dependent. This could be due to the indirect interaction of MRTF mediated and YAP mediated transcription pathways for mechanotransduction of fibroblasts 105. It was found that disruption of transcription coactivator p300 through shRNA mediated knockdown, or a p300 inhibitor abolishes stiffness-induced HSC activation 106. Wang et al. demonstrated that under TGFβ1 stimulation p300 was bound to TAZ and transported it to the nucleus of HSCs. In HEK cells, YAP activity was promoted by overexpression of HA-tagged p300 and its related protein CREB- binding protein (CBP) 107, 108. Manneart and co-workers transfected HSCs with YAP siRNA in 3D aggregates and then transferred them into plastic dishes after 4 days, resulting in their inhibition 109.

## LSECs and YAP

Liver sinusoidal cells (LSECs) are another non-parenchymal cells that play an essential role in liver injury by influencing regeneration and fibrosis through angiocrine signalling to stellate cells in case of acute and chronic liver damage 110. Mechanosignaling in LSECs is induced by shear stress (caused by blood flow), stimulating them to release angiocrine factors that contribute to the development and maintenance of liver function 111. For instance, subjecting LSECs to shear stress results in activation of β1- integrin, as well as vascular endothelial growth factor receptor 3 (VEGER3). This, in turn, causes release of hepatic growth factor and triggers proliferation and survival of hepatocytes 112, 113. Under pathological conditions, and especially during fibrosis, mechanotransduction of LSECs thus further accelerates the progression of HCC due to angiocrine and phenotypic changes 114. In case of chronic liver injury, a dysregulated crosstalk between hepatocytes, HSCs and LSECs contributes to liver fibrosis. Differentiated LSECs also maintain quiescence of HSCs, as revealed by co-culture experiments 115. LSECs also maintain cell integrity in single hepatocytes after YAP activation. Angiogenesis in fibrosis is governed by YAP activation in LSECs by means of HIF-1α and VEGF-A expression 116, 117. Moreover, apart from triggering neo-angiogenesis in the diseased liver, it is highly likely that YAP/TAZ play a major role in hepatic blood vessel formation during endothelial cell sprouting and junction maturation alike 118. However, the role of YAP/TAZ signalling in endothelial cells during fibrosis and other liver diseases, remains to be further investigated.

## Interaction of other pathways with the Hippo signalling pathway

Wnts are important proteins that play a major role in controlling many developmental and physiological processes. Their downstream effector is β-catenin, an adapter protein of cadherin type cell-cell adhesion molecules, which activates Wnt target gene expression 119. Since exposure of cells to mechanical alterations obviously influences cell-cell contacts, Wnt-signalling has a mechano-sensitive component. Excessive activation of the Wnt/β-catenin pathway is associated with many types of cancer, including HCC and hepatoblastoma 120. In distinct cell compartments, the Wnt and Hippo signalling pathways interact differently. In the nucleus, cooperation between YAP and β-catenin regulates gene expression e.g. in controlling the heart size, tumor transformation, and maintenance 121. YAP/TAZ regulates phosphorylation of Dishevelled (Dvl), an important component of Wnt/β-catenin pathway, and the nuclear localization of Dvl or β-catenin, thereby inhibiting the Wnt/β-catenin activities 122. However, it is still unknown, whether the mode of interaction between Wnt/β-catenin signalling is limited to and specific for a special cell type. The loss of activity of Mst1/2 in hepatocytes has previously been shown to lead to the activation of Wnt/β-catenin signalling in the liver and, consequently, to formation of liver tumors 123.

Notch signalling is a further pathway interacting with the Hippo pathway. Notch signalling in the liver promotes the formation of oval cells (liver stem cell) 124. Notch signalling is highly activated in HCC patients and the expression of Notch receptor is highly regulated 125. Direct cell-cell contact is needed for the activation of the Notch pathway, allowing the direct Notch receptor to interact with their membrane bound ligands (Jagged and Delta-like) 126. Sequential proteolytic cleavage of notch receptors by the γ-secretase complex and a member of the ADAM family is induced by Notch ligand binding. As a result of this, NICD (Notch intracellular domain) is liberated from the membrane, and enters the nucleus. In the nucleus, it forms a ternary complex with the co-factor RBP-j and participates in the transcriptional regulation of the respective target genes 127. Constitutively expressed hepatic NICD causes liver tumor formation in mice.

Activation of Notch signalling due to loss of activity of Mst1/2 in hepatocytes forms a positive feedback loop with YAP/TAZ, leading to severe liver enlargement and rapid HCC formation 128. There also is a surprising inhibitory role of Wnt/β-catenin signalling to YAP/TAZ activities: in Mst1/2 null mutants, genetic removal of β-catenin in the liver significantly increases the number of tumor nodules. Mechanistically, YAP/TAZ increases the generation of Notch intracellular domain (NICD) by YAP/TAZ 129. Notch signalling inhibits the β-TrCP-mediated degradation of TAZ and stabilizes it by forming a positive feedback loop.

A further layer of complexity is added by the fact that Wnt signalling modulates the cross-talk between Notch and YAP/TAZ: Wnt/β-catenin signalling promotes nuclear localization of DP1 (the dimerization partner of E2F transcriptional factors) through suppressing the positive feedback loop between TAZ and notch, which subsequently inhibits Notch activity 130. Notch inhibition *in vivo* breaks the YAP/TAZ-Notch positive feedback loop and reduces the activity of YAP/TAZ, hepatocyte proliferation and tumor formation 131. Therefore, there is an unexpected function of Wnt/β-catenin signaling in restricting YAP/TAZ and Notch activities that are involved in HCC initiation.

## Conclusion

HCC is generally caused by intensive liver fibrosis characterised by deposition of collagen fibres and a stiffer ECM. Fibrosis leads to activation of various mechanical feedback loops and signalling pathways. One of most prominent and most studied is Hippo signalling pathway or YAP/TAZ pathway. YAP not only contributes to transcription of genes that lead to cellular proliferation but also leads to the activation of HSC and LSECs. YAP is an important protein that plays a major role in mechanotransduction. Furthermore, the Hippo signalling pathway interacts with several other pathways such as Wnt/β-catenin pathway and Notch signalling pathway that further lead to the progression of HCC. Due to the complexity and mutual entanglement of the respective signalling pathways, more focused studies are needed to further establish the relevance of receptor-independent mechanosensing signaling pathways in the development of HCC 132, 133.

From a technical perspective, the role of mechano-signaling in HCC is extremely hard to study in a clinical or *in vivo* setting. Recently, the awareness has grown that 2D cell cultures cannot mimic the exact physiological conditions, especially when mechanical aspects of the ECM are concerned. There is still lack of reliable and easy to handle 3D models for the study of HCC. Consequently, development of valid 3D models, which allow for access to mechanical parameters (e.g. stiffness) as well as to functional analysis with high temporal and special resolution is an important prerequisite.

## Figures and Tables

**Table 1:** A list of genes mechanically regulated in liver disease

Genes mechanically regulated in liver disease	Reference
**Mechanosensing receptors**	
Integrins and Focal adhesions such as Fibronectin, vitronectin and collagens.	42, 117, 118
Adhesion receptors and cell-cell junctions such as cadherins, selectins, and CAMs	119-121
Ion channels such as TREK-1, K+ channel.	122-124
**Hepatocyte nuclear factor 4 alpha HNF4α target genes**	
Baat, F7, Gys2	125, 126
ARP3 actin related protein 3 homolog C (ACTR3C)	127
Tubulin tyrosine ligase like family member 3 (TTLL3)	127
Actin related protein 2/3	42
ARPC3	42
**Genes related to ECM receptor interaction**	
ITGA1, Fibronectin1, Sorting nexin 15, Laminin, Alpha 4	42, 127
**Epithelial cell related genes**	
CLAUDIN 12, RhoA, Src, Β1 Integrin, Phosphorylated FAK	127
**Other expression of HCC genes**	
CXCL12, IL11, IL6, PDGFA, PDGFB, VEGFA	5, 22, 41, 128
YAP1	90, 91
Megakaryoblastic leukemia factor-1 (MKL 1) (called as myocardium related transcription factor)	94
**Genes involved in mechano signalling of LSECs**	
Β1- Integrin, Vascular endothelial growth factor (VEGFR3), CXCL1	53, 99, 100

**Table 2:** A list of genes regulated by YAP in HCC

YAP regulated genes in HCC	Mode of regulation	Reference
**TEAD Transcription factor family** (play key roles in normal cell growth. N-terminal region of YAP interacts with C-terminal region of TEAD protein).		
RUNX2	Downregulated	129
ITGB2	Upregulated	130
ERBB4	Upregulated	131
CYR61	Downregulated	133
CTGF	Upregulated	132
AREG	Downregulated	134
MYC	Upregulated	135
Gli	Upregulated	136
Vimentin	Upregulated	137
AXL	Upregulated	138
**P73** (major tumor repressor protein. YAP acts as a transcriptional co-activator of P73).		
BAX	Upregulated	139
PIG3	Downregulated	140
c-ABL	Upregulated	141
P53AIPI	Upregulated	142
**ERBB-4** (EGFR family member receptor protein tyrosine kinase translocated in nucleus functioning as transcriptional regulator. It acts as a binding partner for YAP and TEAD. YAP-ERBB4 regulates organ size and tissue growth by promoting expressions of below mentioned genes).		
CTGF	Upregulated	143
CYR61	Downregulated	133
ANKRD1	Upregulated	137
**EGR1** (a nuclear protein functioning as a transcriptional regulator. It interacts with YAP and via PPXY motif of EGR-1 and WW domains induces the expression of BAX).	Downregulated	144
**TBX5** (YAP, β-catenin and TBX5 forms a complex to induce the expression of transcriptional targets for cancer cell survival and transformation).		
P300	Upregulated	144
PCAF	Downregulated	140
**SMADs** (intracellular proteins that transduce extracellular signals from TGF-β or BMP to the nucleus, activating transcription of downstream target genes. YAP/TAZ acts as a regulator of TGF- β-SMAD signalling).		
SMAD2/SMAD3	Upregulated	145
**RUNXs** (members of DNA -binding transcription factor that act as regulators of development. RUNXs interact with YAP and play critical role in regulating cytoskeletal gene expression).		
RUNX1/RUNX2/RUNX3	Upregulated	146
